# Stabilization of the SNARE Core by Complexin-1 Facilitates Fusion Pore Expansion

**DOI:** 10.3389/fmolb.2021.805000

**Published:** 2021-12-14

**Authors:** Josh Pierson, Yeon-Kyun Shin

**Affiliations:** Professor Yeon-Kyun Shin Lab, Roy J. Carver Department of Biochemistry, Biophysics and Molecular Biology, Iowa State University, Ames, IA, United States

**Keywords:** SNARE, single-molecule, fusion pore, compelxin-1, TIRF

## Abstract

In the neuron, neurotransmitter release is an essential function that must be both consistent and tightly regulated. The continuity of neurotransmitter release is dependent in large part on vesicle recycling. However, the protein factors that dictate the vesicle recycling pathway are elusive. Here, we use a single vesicle-to-supported bilayer fusion assay to investigate complexin-1 (cpx1)’s influence on SNARE-dependent fusion pore expansion. With total internal reflection (TIR) microscopy using a 10 kDa polymer fluorescence probe, we are able to detect the presence of large fusion pores. With cpx1, however, we observe a significant increase of the probability of the formation of large fusion pores. The domain deletion analysis reveals that the SNARE-binding core domain of cpx1 is mainly responsible for its ability to promote the fusion pore expansion. In addition, the results show that cpx1 helps the pore to expand larger, which results in faster release of the polymer probe. Thus, the results demonstrate a reciprocal relationship between event duration and the size of the fusion pore. Based on the data, a hypothetical mechanistic model can be deduced. In this mechanistic model, the cpx1 binding stabilizes the four-helix bundle structure of the SNARE core throughout the fusion pore expansion, whereby the highly curved bilayer within the fusion pore is stabilized by the SNARE pins.

## Introduction

**FIGURE 1:**
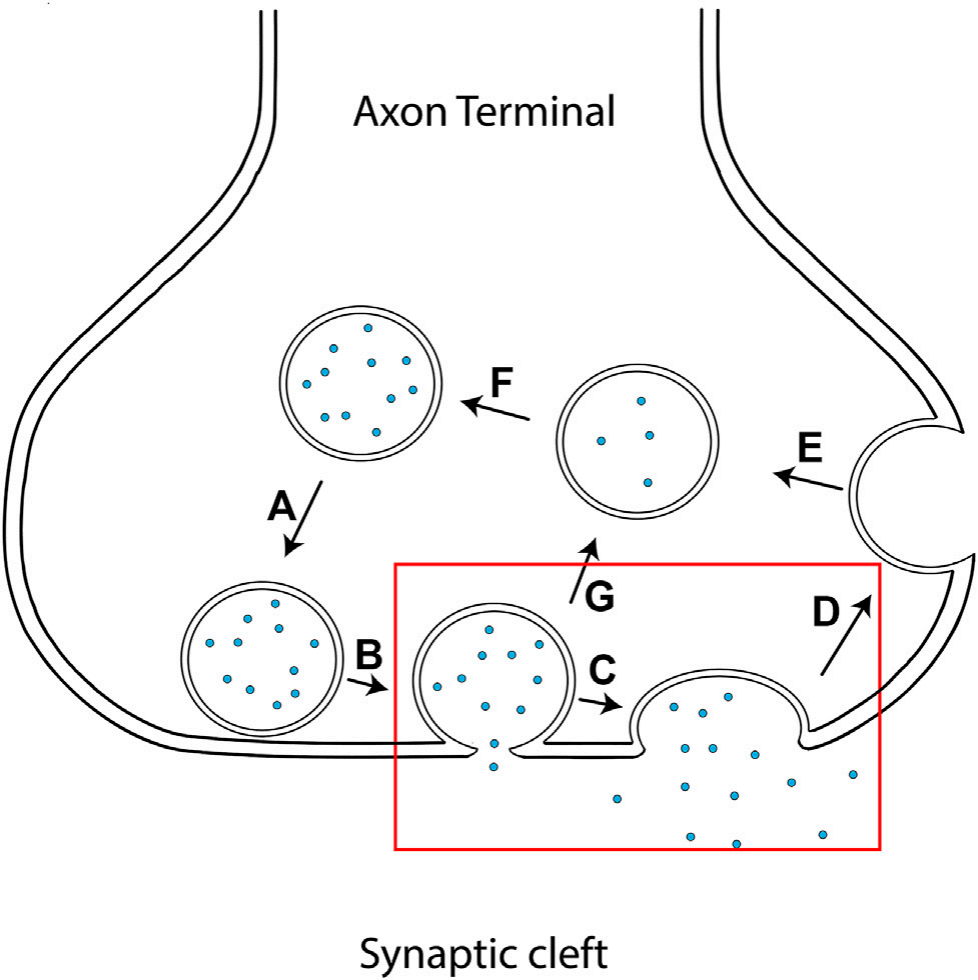
Schematic diagram of vesicle fusion and recycling at the axon terminal. **(A)** Vesicle approaches the plasma membrane. **(B)** Vesicle engages in membrane fusion resulting in a fusion pore, thereby releasing some neurotransmitters. **(C)** The pore expands and the vesicle completely merges with the plasma membrane. **(D)** The cell replenishes its vesicle pool by endocytosis forming a new, unfilled vesicle. **(E)** The newly formed vesicle is filled with neurotransmitters. **(F)** The filled vesicle is brought back into the pool of available vesicles for subsequent rounds of vesicle fusion. **(G)** Rather than entering the pathway in **(D)**, the vesicle engages in a “kiss-and-run” event in which some neurotransmitters are allowed to be released before closing. The vesicle is then detached from the presynaptic membrane and recycled into the presynaptic vesicle pool.

Neurons are the foundation for many fundamental processes in the human body. The central nervous system governs movement, sensory, cognition, and memory. The neurons that compose the nervous system must be able to communicate with each other in a highly regulated manner to facilitate the exquisite orchestration necessary for these essential processes. They execute this via neurotransmitter release into the synapse. The pre-synapse engages in an important mechanism called vesicle fusion in which vesicles, containing neurotransmitters, fuse with the plasma membrane. This fusion allows neurotransmitter release into the synaptic cleft so that they can transmit a signal from one neuron to the next *via* binding to receptors on the post synaptic membrane. Without vesicle fusion, communication among neurons would halt, thus pointing to the importance of the membrane fusion mechanism and machinery. The core machinery essential for this process is composed of three proteins making up the SNARE (soluble N-ethylmaleimide-sensitive factor (NSF) attachment protein receptor) complex (Sӧllner et al., 1993; Weber et al., 1998). Two proteins are located on the target plasma membrane (t-SNAREs); a membrane imbedded syntaxin-1 and a peripheral prenylated SNAP-25. The third member called VAMP2 (v-SNARE) is located on the synaptic vesicle. As the v-SNARE associates with the t-SNAREs, a parallel 4-stranded coiled-coil forms to bridge the two membranes (Poirier et al., 1998; Sutton et al., 1998). The zippering from the membrane-distal N-terminal region to the membrane-proximal C-terminal region (Gao et al., 2012; Min et al., 2013; Shin et al., 2014) provides the energy needed to overcome the energetic barrier for merging the two leaflets. Membranes are then remodeled through hemifusion (Lu et al., 2005; Xu et al., 2005) which is then followed by an initial small fusion pore through which neurotransmitters can be released (Breckenridge and Almers 1987; Han et al., 2004). This fusion pore may then expand and result in full vesicle fusion (Figure 1) (Chernomordik and Kozlov, 2003). Yet, there is another pathway a vesicle could follow to be recycled. Instead, after a short release, the fusion pore may reversibly seal which would result in vesicle disengagement from the plasma membrane. This is termed “kiss and run” (Alabi and Tsien, 2013). These two pathways offer alternative mechanisms of vesicle recycling. In full fusion, new vesicles will need to be reformed though endocytosis from the plasma membrane and filled with neurotransmitters. In the latter, the vesicle remains in an original form and could re-engage in vesicle fusion after being refilled with neurotransmitters. The protein factors and mechanisms controlling these two mechanisms have been elusive and remain unknown. Here, we explore the possibility that complexin-1 (cpx1) contributes to vesicle recycling pathways.

Complexins are a family of small soluble protein (14 kDa) composed of an unstructured N-terminus, two *a*-helical domains (accessory domain and core domain, respectively) followed by a long, unstructured C-terminus. When complexin-1 (cpx1) and -2 (cpx2) are knocked down, both an increase of spontaneous release and a decrease of synchronous Ca^2+^-triggered release have been observed (Maximov et al., 2009). Thus, these two unique roles have been attributed to cpxs. The exact mechanisms by which cpxs engage in inhibition of spontaneous release has been debated for years (Trimbuch et al., 2014). The prevalent explanation is a clamping mechanism in which cpx1 inserts itself into the SNAREpin (Giraudo et al., 2006; Schaub et al., 2006; Tang et al., 2006). This freezes SNARE zippering and spontaneous vesicle fusion. The cpx1 clamp is then removed by the Ca^2+^ activation of synaptotagmin 1 (syt1), which, in turn, displaces cpx1 and allows for fusion (Giraudo et al., 2006; Schaub et al., 2006; Zhou et al., 2015). However, it has been found that syt1 may concurrently bind the SNARE complex with cpx1 (Zhou et al., 2017).

For cpx1, the N-terminal domain is thought to mediate Ca^2+^-synchronized, fast exocytosis, while the helical accessory domain might play an inhibitory role in the absence of Ca^2+^ (Xue et al., 2007; Maximov et al., 2009; Xue et al., 2010; Trimbuch and Rosenmund, 2016). The core domain is responsible for binding to the SNARE complex, which is essential for all functional roles of cpx1 (Chen et al., 2002).

Structural studies have revealed that the core domain of cpx1 is able to bind the SNARE complex in an anti-parallel fashion (Chen et al., 2002). The core domain stabilizes the VAMP2/syntaxin-1A interface which may be compromised by ionic repulsions from the lipids incorporated in the vesicle and target membrane (Chen et al., 2002). This additional stability may contribute to the enhancement of Ca^2+^ evoked release when cpx1 is present. In addition, the C-terminal domain of cpx1 has been shown to have membrane binding properties which could assist in vesicle priming. A mix of both hydrophobic and positively charged residues next to an amphipathic region may allow a motif of the C-terminal region to bind to membranes either disorderly, or as a *a*-helical form (Snead et al., 2014). Furthermore, this binding motif seems to localize to highly curved membranes (Snead et al., 2014; Gong et al., 2016).

To investigate cpx1’s role in fusion pore expansion and ultimately, its role in vesicle recycling, we employ an *in vitro* single vesicle-to-supported bilayer fusion assay (Liu et al., 2005; Khounlo et al., 2021). This assay allows us to monitor, in real time, cpx1’s effect on SNARE mediated fusion pore expansion. By using a 10 kDa polymer as our probe (∼6 nm in diameter), we are able to observe the formation of a large fusion pore in a single vesicle fusion event one at a time. Interestingly, we observe that wild-type (WT) cpx1 drastically increases the probability of large pore formation through SNARE binding. We find that it is the core domain of cpx1 that is mainly responsible for the increased large pore formation.

## Results

### Complexin-1 Promotes Large Fusion Pore Formation

Extensive research has been carried out to understand the functions of cpx1 in vesicle fusion using knock down, deletion, and *in vitro* fusion assays (Trimbuch and Rosenmund 2016). However, studying cpx1 function in SNARE-dependent vesicle fusion with an *in vitro* vesicle-to-bilayer content assay can be highly beneficial. In fact, we are able to dissect progression of the fusion pore in real time by differentiating between small and large pore formation. Moreover, this *in vitro* assay allows us to investigate cpx1/SNARE specific interactions in the absence of other protein factors and under controlled conditions.

In the single vesicle-to-supported bilayer fusion assay, we use flow cells to inject vesicles encapsulating our chosen dye onto a preformed supported bilayer. The vesicles and the supported bilayer have been reconstituted with VAMP2 and t-SNAREs (Syntaxin-1A and SNAP-25), respectively. The planar proteo-bilayer is suspended above a hydroxylated quartz slide by PEG thus, creating a PEG-pillared aqueous gap in which our polymer fluorophore can diffuse into once it is released from the vesicle.

To image, we utilize total internal reflection fluorescence (TIRF) microscopy. TIRF relies on an incident beam at a specific angle (exceeding the critical angle) between two mediums of different refractive indices to create an evanescence wave within approximately 100 nm on the surface. The light intensity decays exponentially as distance gets further from the interface. This gives us the unique ability to, at least qualitatively, decipher fluorophores’ distance based on how bright of a signal we receive. As our vesicles flow through the chamber, we do not see any stationary vesicles other than bump-and-run vesicles unless they directly interact with the bilayer. Then, as the vesicle approaches the bilayer, they start to fluoresce.

**FIGURE 2:**
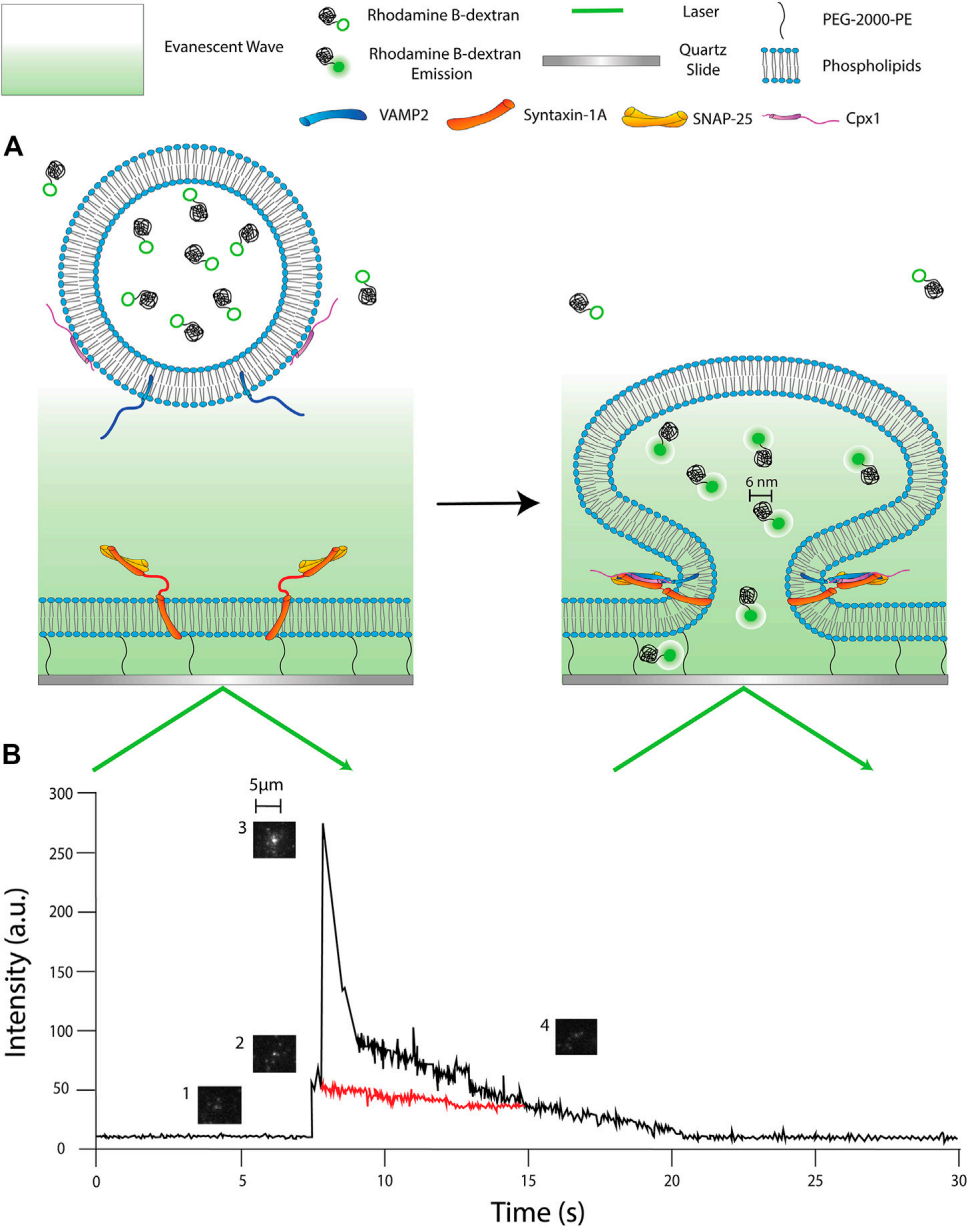
Schematics of single vesicle-to-supported bilayer fusion assay. **(A)** **(Left panel)** the reconstituted vesicle is filled with Rhodamine B dextran (RB-dextran) and has not yet entered the region of the evanescent wave. **(Right panel)** the vesicle has engaged with the supported bilayer and formed a pore larger than ∼6 nm; the RB dextran is fluorescent and diffuses into the PEG-pillared aqueous space. **(B)** A fluorescence time-trace of a single fusion event. 1) The vesicle approaches the membrane but has not entered the evanescent wave. 2) The vesicle docks, resulting in an increase in fluorescent intensity. 3) The fusion pore expands to a larger diameter, causing a large spike in fluorescence. 2D diffusion of dye is being observed. 4) The pore constricts, but the internal content slowly diffuses out of the vesicle. (Red trace) Exemplary time trace of an event without achieving a large fusion pore status. The trace does not show an early fluorescence spike nor 2D diffusion.

We have chosen Rhodamine B conjugated to 10 kDa dextran (RB-dextran) as our fluorophore. Previously, we have used an 11 kDa fluorescent DNA probe to detect fusion pore expansion in the single vesicle-to-vesicle fusion. Thus, we have chosen 10 kDa RB-dextran as an extension of our previous studies of the fusion pore (Diao et al., 2010; Lai et al., 2013). The hydrodynamic radius of the 10 kDa polymer conjugated to our Rhodamine B is ∼6 nm. This has two important implications. First, this slows down the rate of 2D diffusion so we are able to visualize it at the speeds our camera can capture (a few msec). Second, this allows us to gauge the diameter of the pore. Since the hydrodynamic diameter of the polymer is estimated to be ∼6 nm, we can detect the presence of a single large fusion pore when we detect fluorophore 2D diffusion from the vesicle. This is one of the benefits of using this particular single molecule technique. We are able to determine whether or not a vesicle has reached a large pore status because we can microscopically see the individual fluorophores diffusing from the fusing vesicle (Figure 2A).

In our analysis, we can dissect each fusion event we capture one by one. We can monitor if a large fusion pore is present and for how long. Additionally, the fluorescence intensity time trace (Figure 2B) tells us real-time behavior of individual vesicles during membrane fusion. In general, the time trace has an initial sudden increase of intensity as the vesicle comes into contact with the bilayer and docks. However, as the fusion pore forms and expands, we expect the shape of the vesicle to deform in such a way that the dye inside the vesicle is brought closer to the surface and deeper into the evanescent layer. This would result in an increase in fluorescence intensity (Khounlo et al., 2021). Once the fusion pore reaches a critical diameter (∼6 nm), it then allows the release of the dyes from the vesicle into the gap between the bilayer and the quartz surface. The diffusion of the dyes through the interspace gap can be visually observed through the microscope. Concurrently, the fluorescence intensity at the center decreases as the dyes move away from the fusion site.

In most cases, large pores, with time, constrict prematurely. This constriction might leave a small fusion pore and a solid fluorescence spot that fades out until the fluorescence intensity eventually returns to baseline. We speculate this event to be a slow leakage of the content through a small fusion pore.

Khounlo and coworkers, using the same technique, probed the internal content release and large pore formation in SNARE-only case (Khounlo et al., 2021). It was found that only approximately ∼38% of the docked vesicles advanced to the large pore stage while ∼62% demonstrated small or no pore formation, which results in no internal content release. When we repeated the assay under the SNARE only conditions, we found that out of 170 events, 47% (80 events) advanced to a large pore state, while 53% (90 events) did not develop the large fusion pore. Thus, we have been able to confirm the previous finding that with SNAREs only, the majority of docked vesicles do not advance to the large pore states. We then used this approach to investigate the effect that cpx1 has on SNARE-mediated fusion pore formation and expansion.

Cpx1 can both inhibit or stimulate vesicle fusion depending on its concentration. At high concentrations, the inhibitory function dominates, while, at lower concentrations, the stimulatory function dominates (Yoon et al., 2008). Concentration-dependent curves for vesicle-vesicle content mixing with cpx1, syt1, and Ca^2+^ show that at around 200–300 nM, cpx1 has the highest capability to enhance content mixing. However, after 200–300 nM, cpx1 enhancement decreases gradually and eventually shifts to an inhibitory role (Kim et al., 2016). Our *in vitro* assay allows us to focus on the stimulatory aspect of cpx1 by selectively applying the specific cpx1 concentration. Thus, we chose to use a concentration of 300 nM cpx1, as it best enhances SNARE-dependent vesicle fusion.

Previously, Weninger and coworkers measured the on- and off-rates of cpx1 to the membrane-bound SNARE complex using single molecule fluorescence resonance energy transfer (FRET). They found that the single molecule dissociation constant Kd for this interaction is ∼43 nM. Thus, at 300 nM cpx1, we expect that almost all SNARE complexes are bound to cpx1 (Li et al., 2007).

Prior to injection, we incubated both the reconstituted vesicles and the reconstituted supported bilayer separately with 300 nM of cpx1 for 10 min. This was to ensure that the concentration of cpx1 is constant throughout our measurements. We then flowed the mixed vesicle solution into the flow channel and recorded the membrane fusion events in real time.

**FIGURE 3:**
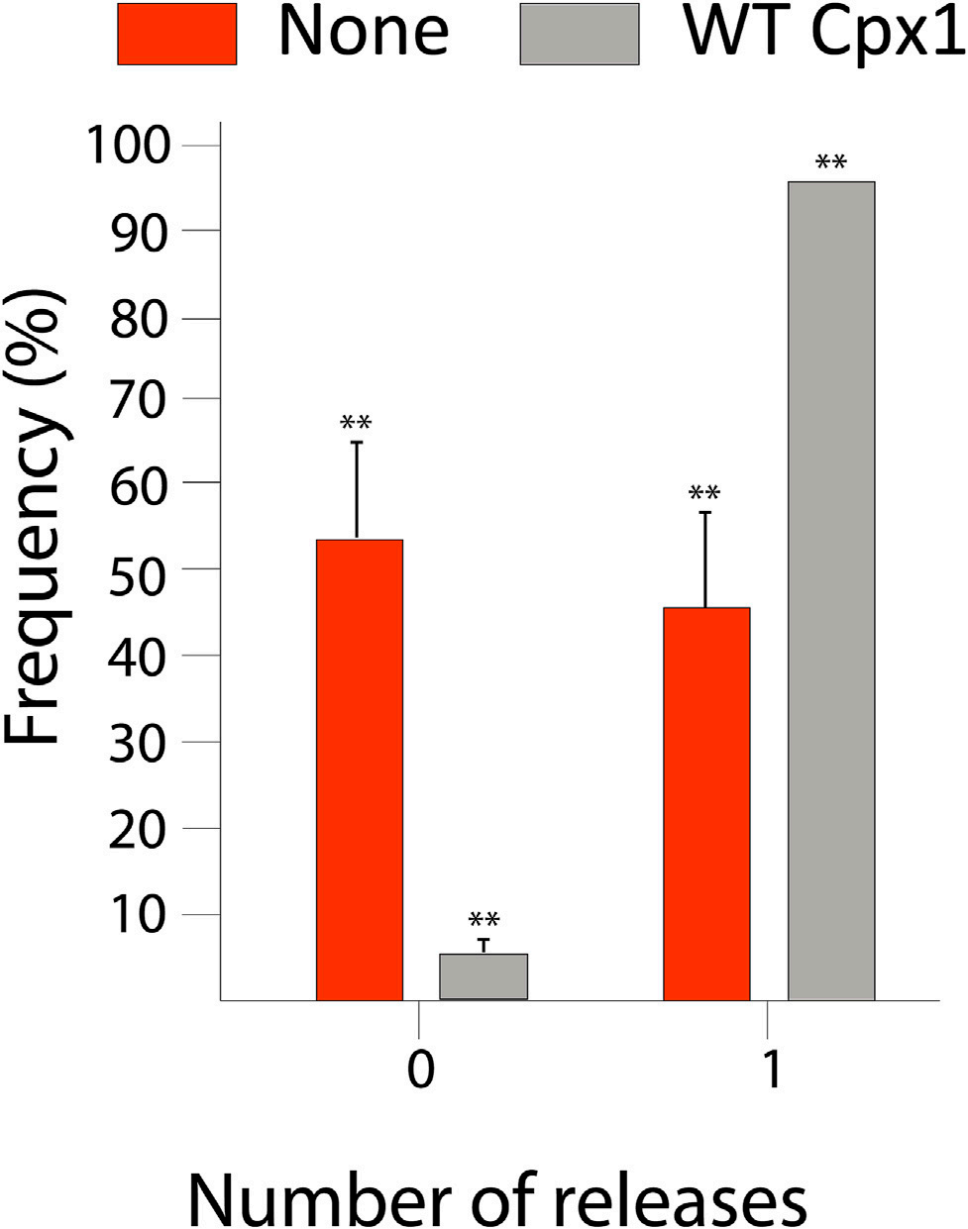
Wild-type (WT) cpx1 increases the probability of large fusion pore formation. Bar graph displays the percent distribution of large fusion pore forming events among docked vesicles. The data is shown as means ± SD. **p ˂ 0.01 by Students’ t-test; *n* = 3 independent experiments.

Our analysis yielded that out of 432 total events, 412 (95%) showed large pore formation. Whereas, only 20 of them (5%) did not show large pore formation (Figure 3). Thus, the results show that cpx1 increases the likelihood of large pore formation. This is consistent with our previous work using the 11 kDa DNA FRET probe in the single vesicle-to-vesicle fusion assay, where we found that 5 µM cpx1 showed the stimulation of fusion pore expansion (Lai et al., 2013). Our results suggest that cpx1 may have an impact on whether or not a vesicle engages in a “kiss and run” fusion event or a full fusion event at the synapse and favor the full fusion pathway.

### SNARE-Binding Core Domain of Complexin-1 is Responsible for the Stimulation of Fusion Pore Expansion

**FIGURE 4:**
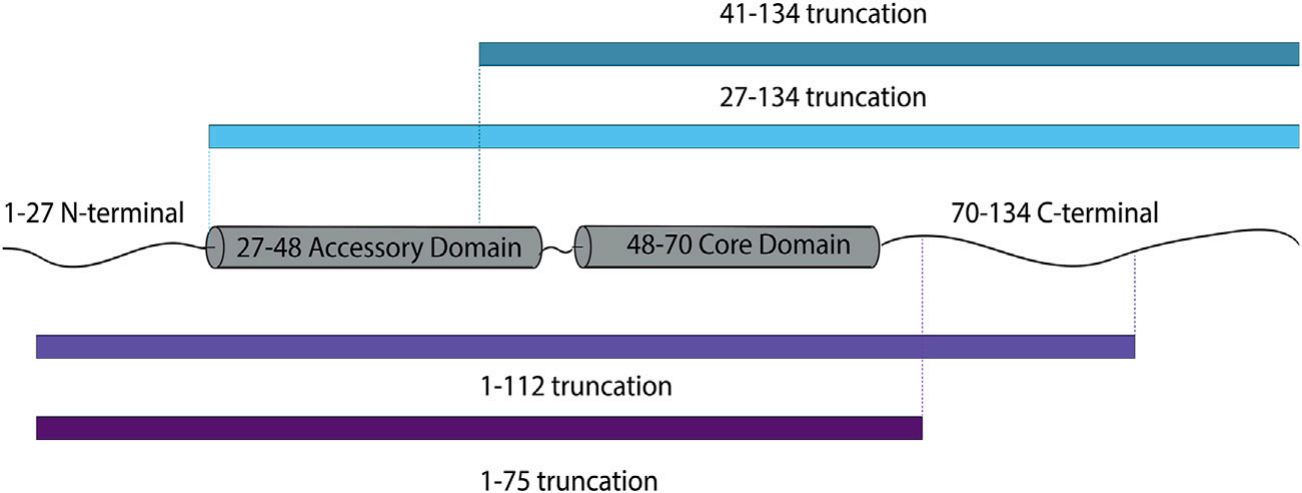
Schematic diagram of cpx1 truncations designed to investigate the effects of individual domains of cpx1 on fusion pore expansion.

Because the dilation of the fusion pore is considered the free energy-demanding step (Chernomordik and Kozlov, 2003), our results might imply that cpx1 assists in overcoming the energy barrier for large fusion pore formation. Here, we dissected cpx1 to establish which domain is responsible for promoting large pore formation. Firstly, a specific region of interest is the C-terminal tail of cpx1 as it was found that this domain preferentially binds to membranes with curvature (Snead et al., 2014; Gong et al., 2016). Because the fusion pore is composed of a highly curved bilayer, this domain has the potential to assist with pore expansion. The C-terminal region of cpx1 is unstructured but, contains the amphipathic, membrane binding domain. Thus, we used a C-terminal truncation mutant (1–112) to remove this particular domain (Figure 4). We also made a complete C-terminal truncation mutant (1–75).

In addition, we wanted to investigate whether or not two N-terminal domains had any implication on fusion pore expansion. We used two N-terminal truncation mutants (27–134) as well as (41–134) in which the first 26 residues of the stimulatory region are removed and the 40 residues, including the inhibitory accessary domain, is removed, respectively (Figure 4).

**FIGURE 5:**
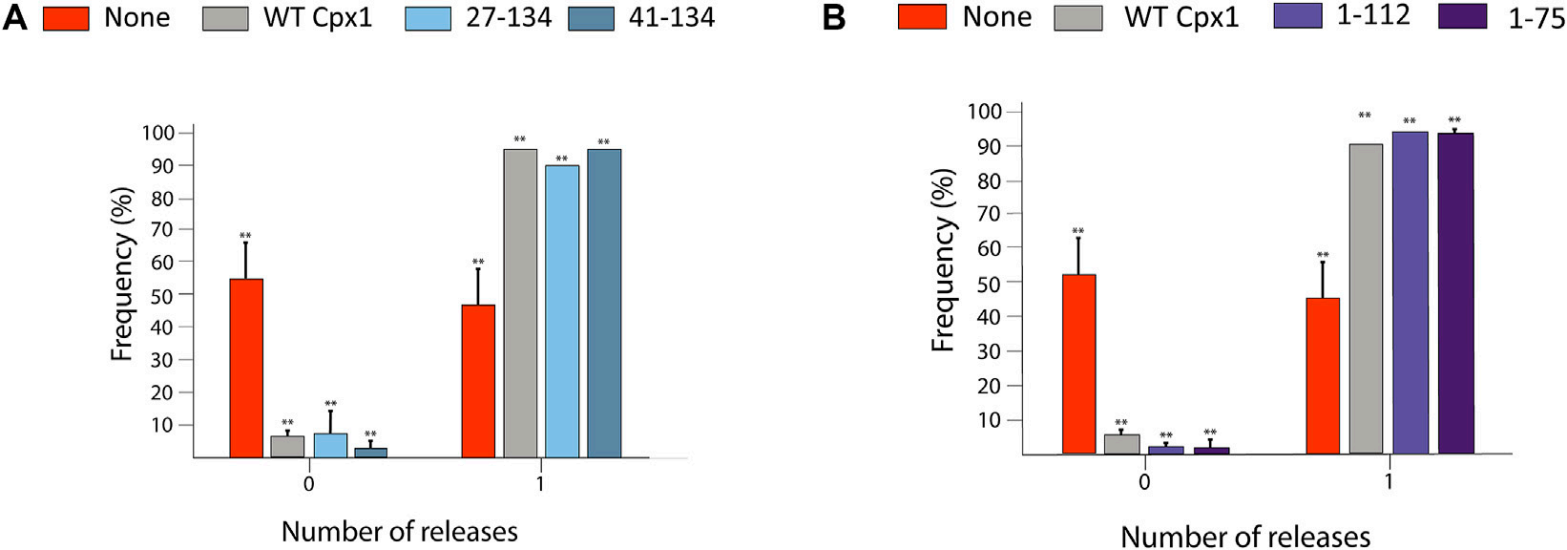
SNARE-binding core domain of cpx1 is responsible for increased probability of large fusion pore formation. **(A)** Bar graph displays the distribution of the percent of vesicles showing large fusion pore forming events among docked vesicles with no cpx1 (red), with WT cpx1, and with N-terminal truncation mutants of cpx1. The data is shown as means ± SD. **p ˂ 0.01 by Students’ t-test; *n* = 3 independent experiments. **(B)** Bar graph displays the percent of vesicles showing the large fusion pore events among docked vesicles without cpx1, with WT cpx1, and with the C-terminal truncation mutants of cpx1. The data is shown as means ± SD. **p ˂ 0.01 by Students’ t-test; n = 3 independent experiments.

We then substituted each of them for WT cpx1, and repeated our experiment as previously described. To our surprise, for the probability of large pore formation, we observed no significant difference between WT cpx1 and our N-terminal truncation mutants (27–134, 41–134) (Figure 5A); nor did we see any significant difference between WT and the two C-terminal truncation mutants (1–112, 1–75) (Figure 5B). Since the core domain is responsible for SNARE binding, this is the only domain that was kept intact for all truncations. All other domain truncation mutants yielded no difference from the WT. Thus, our results show that the core domain is most likely responsible for the increase in the probability of large fusion pore formation by cpx1.

**FIGURE 6:**
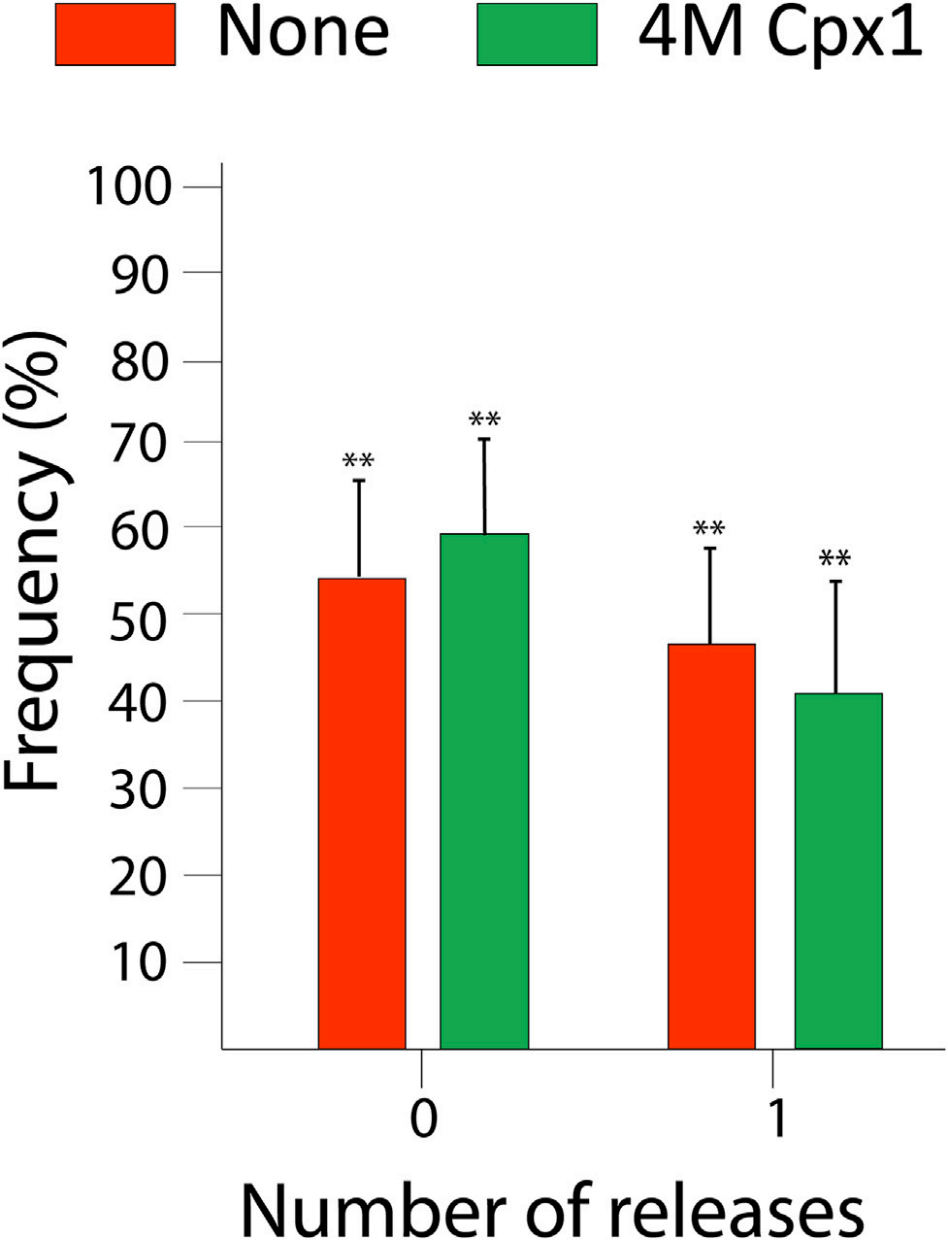
Bar graph displaying the distribution of the percent of vesicles showing large fusion pore forming events among docked vesicles without cpx1 (red) and for the non-SNARE binding cpx1 mutant 4M (green). The data is shown as means ± SD. **p ˂ 0.01 by Students’ t-test; *n* = 3 independent experiments.

To further verify the results, we utilized the 4M mutant which has four essential SNARE binding residues in the core domain mutated to alanine (R48A, R59A, K69A, Y70A). This mutation was previously shown to disrupt SNARE binding to cpx1 (Maximov et al., 2009; Choi et al., 2016). Thus, we expect that the drastic increase of the probability of large pore formation by cpx1 to be significantly reduced with the 4M mutant. When we introduced the 4M mutant to our assay we found that out of 153 events, 62 (41%) exhibited large pore formation and 91 (59%) had no large pore formation (Figure 6). This result strongly supports the conclusion that for cpx1, the SNARE binding of the core domain is responsible for the increased probability of large pore formation.

### Complexin-1 Accelerates Initial Dilation of the Fusion Pore

Our single vesicle-to-supported bilayer fusion assay has allowed us to observe cpx1’s capacity to stimulate large pore formation that could possibly tip the scales in the vesicle recycling pathway. However, this is only part of the data we are able to collect using this method. Here, we present additional data that may help us understand the influence that cpx1 may have on the vesicle recycling pathway. As we have previously described (Khounlo et al., 2021), we are able to make qualitative inferences about the diameter of the pore from the intensity of the fluorescence time trace. As the fusion pore dilates, the vesicle might deform, which leads to a sharp increase in florescence seen on the time trace (Figure 2B). Thus, the more dilated a pore is at the early stage, the higher the florescent intensity will be. We can use the traces and, specifically, the max intensity of each trace to draw qualitative inferences about the size of initially enlarged fusion pores.

To calculate a crude approximation of the relative increase of the fluorescence intensity due to this initial pore expansion, we divided the maximum fluorescence intensity by the intensity right after vesicle docking. Because the docking event is not apparently discernable in many cases, we divided the maximum intensities of individual events by the average of the fluorescence intensities of the immediately docked vesicles.

**FIGURE 7:**
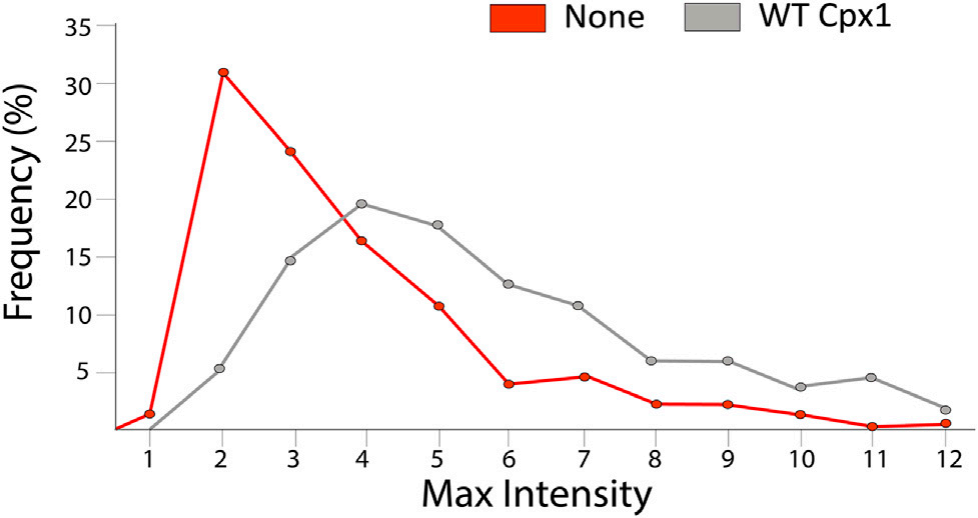
Cpx1 accelerates initial dilation of fusion pore. Distribution of their percent relative max intensity without cpx1 (red) and with WT cpx1 (gray). The relative maximum intensity is calculated by dividing the maximal fluorescence intensity in the time trace by the average fluorescence intensity measured immediately after docking.

With SNAREs only, we found that the maximum intensity increased up to 10 times with an average of a factor of 3.37 ± 2.08 (the median was 2.71) (red in Figure 7). When we analyzed our 432 WT cpx1 large pore events, we found that the distribution became even broader and the average increased to 4.99 ± 2.65 with a median of 4.32 (grey in Figure 7). The increase of the averages (and medians) with cpx1 is further evidence that cpx1 is capable of dilating SNARE-dependent fusion pores. Thus, not only does cpx1 increase the probability of large pore formation, it can also help increase the quick initial expansion of the fusion pore.

### For Truncation Mutants, the Initial Pore Sizes Are Not as Large as That of the Wild-Type Complexin-1

**FIGURE 8:**
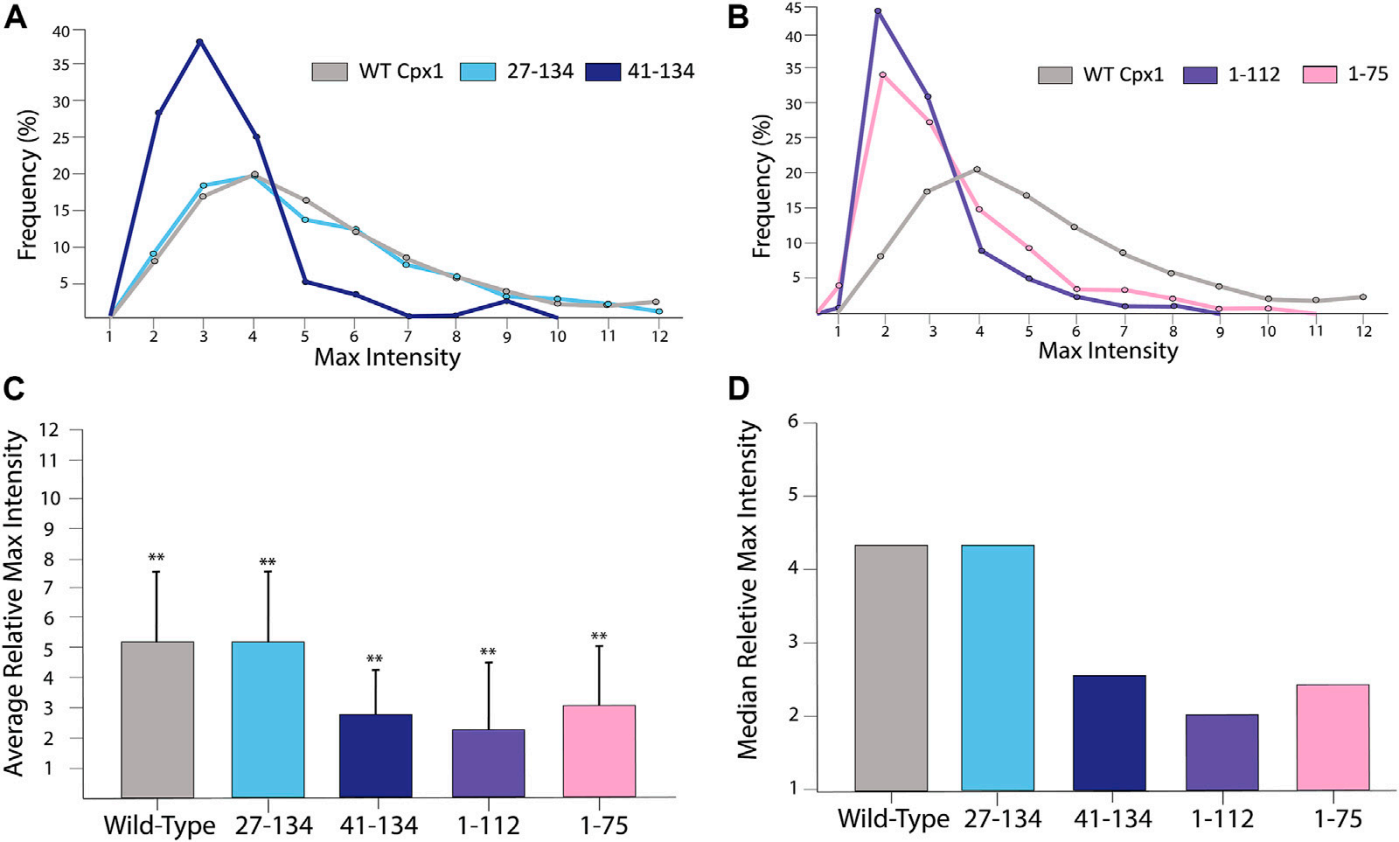
For truncation mutants of cpx1, the pore sizes are not as large as those of the WT cpx1. **(A)** Distributions of the relative maximum intensities of WT cpx1 and the two N-terminal truncation mutants. **(B)** Distributions of the relative maximum intensities of WT cpx1 and two C-terminal truncation mutants. **(C)** Average relative maximum Intensities. The data is shown as means ± SD. **p ˂ 0.01 by Students’ t-test; n = 3 independent experiments. **(D)** Median relative maximum intensities.

To elucidate the domains responsible for the promotion of the initial quick dilation, we then analyzed the relative max intensity for each of the mutations. We first analyzed the N-terminal truncation mutants and found that they did not exhibit any difference from the WT (Figure 8A). This tells us that the N-terminal domain does not have the ability to accelerate initial pore expansion. On the other hand, when analyzing (1–112) we observed that the events had an average relative maximal intensity of 2.39 ± 1.35 with a median of 2.01. Meanwhile (1–75) has an average of 2.99 ± 1.96 and a median of 2.44 (Figure 8B). When compared to the cases of WT cpx1 and SNARE only, we see some loss of initial pore dilating capabilities with both of these truncations.

Additionally, we analyzed the accessory domain truncation (41–134). To our surprise, we observed that the relative maximal intensity dropped to 2.91 ± 1.45 with a median of 2.44 (Figure 8A). Thus, our results show that either the accessary domain (28–40) or the C-terminal domain (113–134) is required for the initial acceleration of fusion pore dilation; although it is unclear if both are required.

### Fusion Pore Size and Duration Are Inversely Correlated

**FIGURE 9:**
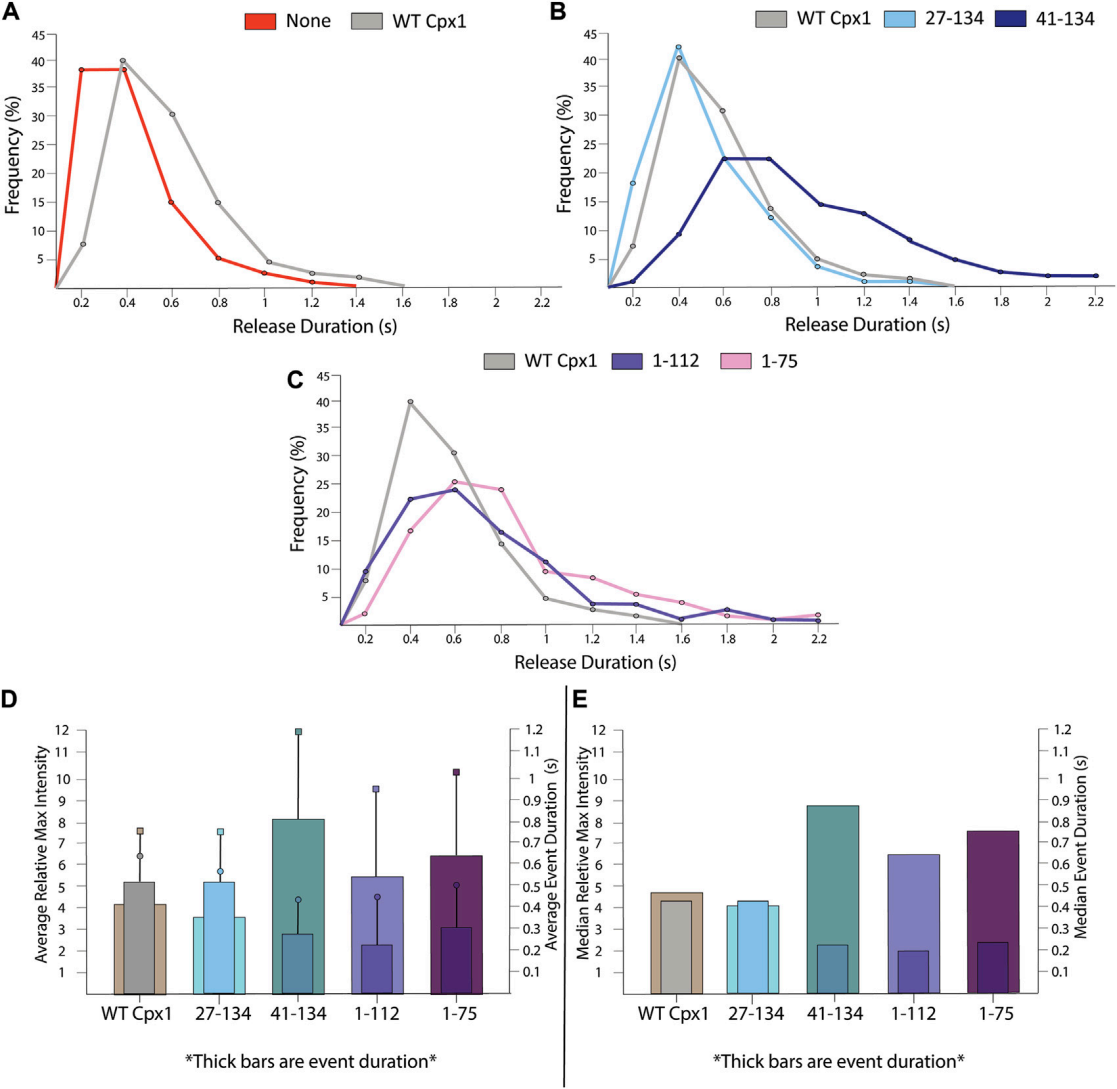
For truncation mutants of cpx1, the duration of the large fusion pore is longer than that of WT cpx1. **(A)** Distribution of release durations without cpx1 and with WT cpx1. **(B)** Distribution of release durations of N-terminal truncation mutants of cpx1. **(C)** Distribution of release durations of C-terminal truncation mutants of cpx1. **(D)** The reciprocal relationship between average relative max intensity **(left *y*-axis)** and average event duration **(right *y*-axis)**. For each graph, the thick bars represent event duration while max intensity is represented by thin bars. Boxes represent SD of event duration. Circles represent SD of relative max intensity. **(E)** The reciprocal relationship between median relative max intensity **(left *y*-axis)** and median event duration **(right *y*-axis)**. For each graph, the thick bars represent event duration while max intensity is represented by thin bars.

The single vesicle-to-supported bilayer fusion assay allows us to track event duration. Event duration is defined as the time period from the appearance of the first fluorophore diffusion, indicating emergence of the large pore, until the time point in which no apparent diffusion is visible. SNARE only data showed an average duration of 0.32 ± 0.21 s. When WT cpx1 was introduced, we found that it had an average event duration of 0.48 ± 0.24 s, which is almost double the duration of the SNARE only event (Figure 9A).

Among the truncation mutants of cpx1, however, we observed some variations in the event duration. We saw that both the WT and the N-terminal truncation mutant (27–141) had nearly the same relative maximal intensities. When comparing their event duration, we saw that, again, there was no significant differences (Figure 9B). For all other mutants, the event duration is slightly longer than that of the WT (Figure 9B,C).

However, we found an interesting correlation between the initial pore dilation and the event duration for the cpx1 mutants. One might expect that the maximum intensity, from which the initial pore dilation is inferred, and the event duration should be inversely correlated. The larger the initial fusion pore is, the faster the internal content be released. Indeed, we found that as pore dilation increases, event duration decreases for both WT cpx1 and the mutants. (Figures 9D,E). While this data is not significant enough to draw standalone conclusions, it helps to supplement our interpretation of our data. As fluorescence intensity increases during an event, we are likely to see an increase in pore dilation and fast content release.

## Discussion

In contrast to the in-depth understanding of vesicle fusion, vesicle recycling mechanisms are poorly understood (Silm et al., 2019). The neuron must have a sufficient number of vesicles ready for fusion at any given time. Even a small variation of the number of vesicles in the releasable pool could be harmful in administering the controlled release of the neurotransmitters. Thus, vesicle recycling is a very important cellular process. Yet, the protein factors that determine whether a vesicle will “kiss and run” or fully fuse remains unclear. In this work, we provide evidence that cpx1 might be one of the important factors that determine the choice of the vesicle recycling pathways.

We observed a dramatic increase of the probability of large pore formation with the addition of cpx1. The analysis with truncation mutants revealed that neither the functional N- or C-terminal domains are responsible for such a promotional role of cpx1 in fusion pore expansion. The results strongly suggest that the binding of the core domain of the cpx1 to the SNARE core contributes to this stimulatory activity towards fusion pore expansion. Consistently, the 4M mutant of cpx1, which abolishes cpx1 binding to SNARE core, negates the promotion of the fusion pore expansion by cpx1. The result that the stabilization of the SNARE core by cpx1 promotes fusion pore expansion is surprising, but an interesting hypothetical mechanism could be inferred from the results.

**FIGURE 10:**
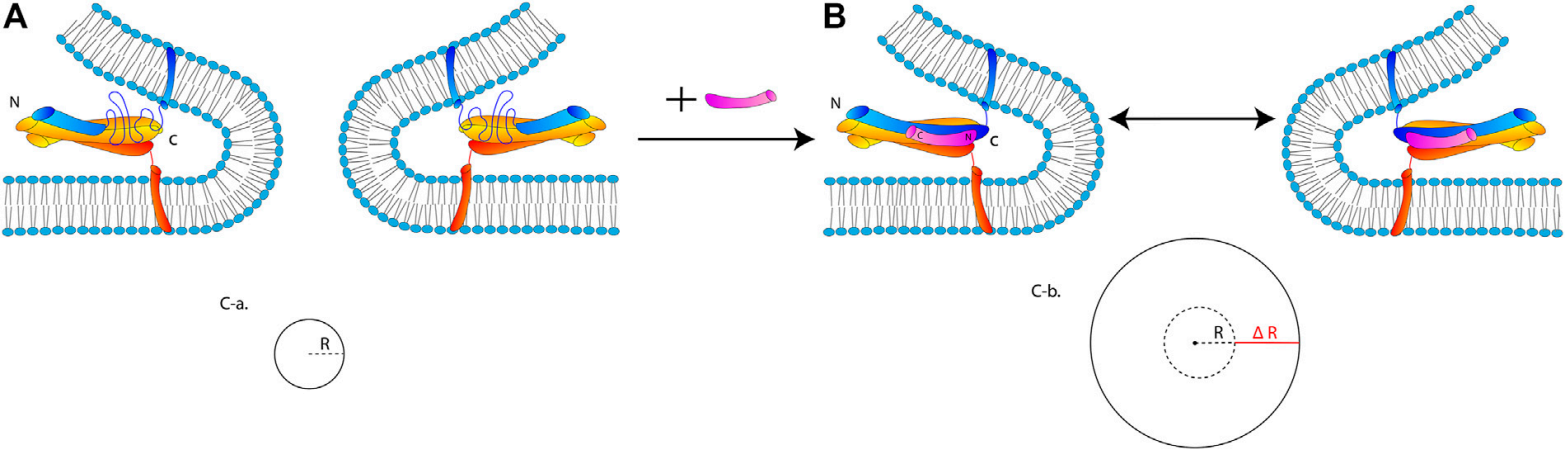
A hypothetical mechanistic model for the promotion of fusion pore expansion by cpx1. **(A)** SNARE complex with the frayed C-terminal half in the absence of cpx1. The SNARE complex is unable to overcome the curvature tension and thus, VAMP2, at the c-terminal, becomes dislodged and unraveled; thus, resulting in a failure to advance to the large fusion pore. **(B)** Robust SNARE four-helix bundle stabilized by cpx1 pins two regions of the bilayer flanking the highly curved fusion pore. **(C-a)** The small pore is represented by the circle with the radius R. **(C-b)** The small pore has expanded by ΔR with the addition of cpx1 because less energy is required for fusion pore expansion due to the stabilization of the curvature by the SNARE-cpx1 pin.

In Figure 10, we propose a hypothetical mechanism by which the stabilization of the SNARE core by cpx1 stimulates expansion of the fusion pore. In this mechanistic model, cpx1 binding to the membrane-proximal C-terminal region of the SNARE core keeps the four-helix bundle intact through fusion pore expansion, which then stabilizes the expanding fusion pore.

The free energy associated with vesicle fusion increases with each stage. Hemifusion has a smaller energy barrier, pore formation has a greater energy barrier, and the expansion of that fusion pore has the greatest energy barrier and thus, is the rate limiting step (Chernomordik and Kozlov, 2003). The fusion pore is composed of a tightly curved bilayer with high curvature tension. The expansion of the fusion pore requires an increase of the curved bilayer by 2πΔR, where ΔR is the increase of the radius of the fusion pore (Figure 10C). Thus, the free energy ΔG required for pore expansion should be proportional to 2πΔR, which must be overcome to drive the fusion pore expansion.

By keeping the complete four-helix bundle intact throughout the pore expansion with cpx1, the free energy ΔG required for the expanding fusion pore would be significantly lowered. This is because the two bilayer regions creating the highly curved fusion pore are fastened by the SNARE core-cpx1 complex. In contrast, in the absence of cpx1, the C-terminal half of the SNARE core may not be sufficiently robust to overcome the curvature tension. In fact, it is previously shown that the C-terminal half of the SNARE core is soft and VAMP2 in this region could be easily be dislodged from the four-helix bundle with a small force (Min et al., 2013; Gong et al., 2016). If so, the newly formed small fusion pore would be less likely to expand to a larger size, which is fully consistent with our experimental observation.

Cpx1’s ability to increase pore expansion probability can help determine the recycling pathway of a vesicle. “Kiss-and-run” behavior allows for a quick and small release of neurotransmitters, which can be beneficial for the cell because the vesicle does not need to be reformed. Yet, there are many times in which there is cause for a complete release of the vesicle cargo to guarantee the signal is properly transduced. Our data indicates that cpx1 may be one of the proteins the cell can utilize in this fashion. Cpx1’s unique ability to not only bind the SNARE complex, but also stabilize the complex, makes cpx1 a prime candidate for cells to employ to increase the likelihood that the incoming signal is passed onto the next neuron through the complete vesicle fusion pathway.

In this study we have investigated cpx1’s effect on pore formation and expansion. We have found that cpx1’s core domain is able to promote SNARE mediated pore expansion by maintaining the intact four-helix bundle structure throughout the process.

## Materials and Methods

### Plasmid Constructs and Complexin-1 Mutagenesis

SNAP-25 (aa 1–206), synatxin-1A (aa 1–288), VAMP2 (aa 1–116), WT cpx1 (aa 1–134) and truncations (27–134), (41–134), (1–75), and (1–112) are inserted into pGEX-KG vectors with N-terminal glutathione S-transferace (GST) fusion proteins as a tag. 4M cpx1 mutant (R48A, R59A, K69A, Y70A) was inserted into a pET-28b vector containing a C-terminal 6 Histidine-tag. All site-directed mutations/truncations were performed using promoters supplied by Iowa State DNA facility. All sequences confirmed by Iowa state University DNA Sequencing Facility.

### Protein Expression and Purification

All N-terminal GST fusion proteins (SNAP-25, synatxin-1A, VAMP2, WT cpx1, and all cpx1 truncation mutants) were expressed in *Escherichia coli* BL21 (DE3) competent cells. Cells were then grown in LB medium with ampicillin (100 ug/mL) at 37̊°C until an OD absorbance of 0.6–0.8 (600 nm) was reached. The cells were allowed to cool to stop division and then induced using IPTG (isopropyl *ß*-d-thiogalactopyranoside, 0.3 nM for final concentration) overnight at 16°C. They are then pelleted at 4,000 rpm for 15 min and resuspended in a wash solution (497 mM NaCl, 2.7 mM KCl, 10 mM Na_2_HPO_4_, 1.8 mM KH_2_PO_4_, pH 7.4, [4 g/L Triton-X 100 added for the membrane proteins, VAMP2 and syntaxin-1A]) with final concentrations of 1 mM AEBSF [4-(2-aminoethyl) benzenesulfonyl fluoride and 4 mM DTT. The cells are homogenized to lyse and then centrifuged at 15,000 rpm for 30 min to separate the supernatant from the pellet. The supernatant is equilibrated with glutathione resin for 2 h to allow for maximal GST binding. The resin is then washed using wash solution to remove all things unbound then equilibrated with the elution buffer (137 mM NaCl, 2.7 mM KCl, 10 mM Na_2_HPO_4_, 1.8 mM KH_2_PO_4_, pH 7.4, [0.8% octyl-beta-glucoside (OG) was added for the membrane proteins]). To cleave the GST tag, 25 U of thrombin was added and incubated at 4°C for 12 h. After elution, AEBSF was added to stop thrombin cleavage, 15% glycerol was mixed in, and the protein was stored in −80 C.

The 4M mutant was purified using a His-tag. The protein was expressed similarly, however, the antibiotic used was kanamycin (50 μg/ml). The cells were induced, pelleted, resuspended, and lysed in the same manner. The supernatant was equilibrated with Ni-NTA resin. The protein was then washed using the wash buffer with 5 mM of imidazole. The protein was removed from the resin using the elution buffer described and 400 mM imidazole. The imidazole was dialyzed out overnight. 15% glycerol was then added and the protein was stored at −80 C.

### Lipid Preparation

The supported bilayer was made from POPC (1-palmitoyl-2-dioleoyl-sn-glycero-3-phosphatidylcholine), DOPS (1,2-dioleoyl-sn-glycero-3-phosphatidylserine), PIP2 (phosphatidylinositol 4,5-bisphosphate), and PEG2000-PE (1,2-dipalmitoyl-sn-glycero-3-phosphoethanolamine-N-[methoxy (polyethylene glycol)-2000]) in chloroform at a molar ratio of 78:15:2:5. The lipid mixture (t-lipid) was mixed and then dried using an air stream until the lipid mixture coated the side of the glass vial. Afterwards, the vials were put into a vacuum overnight. The t-lipids were resuspended in HEPES-OG buffer (25 mM HEPES/KOH, 150 mM KCl, 1% *ß*-OG, pH 7.4).

A separate lipid mixture (v-lipids) that would become vesicles were comprised of POPC, DOPS, and cholesterol at a molar ration of 75:5:20. The v-lipids are dried and vacuumed overnight similarly to the t-lipids. Then, v-lipids are resuspended in HEPES with 90 μM of Rhodamine B conjugated to 10 kD dextran (RB-dextran). The resuspended lipids are subjected to 10 flash freeze-that cycles in liquid nitrogen and boiling water, respectively. They were then formed into unilamellar vesicles by extrusion though 100 nm diameter polycarbonate filters and the v-liposomes were stored on ice.

### SNARE Reconstitution

To prepare the supported bilayer, syntaxin-1A and SNAP-25 are premixed for a half hour at room temperature at a molar ratio of 1:1.5 in order to form the t-SNARE complex. The t-lipid is then added to the mixture at lipid: syntaxin-1A ratio of 2000:1 and allowed to incubate for another 10 min. The mixture is diluted, using HEPEs buffer, 3-fold to reduce the concentration of detergent and insert the transmembrane domain into the t-lipids. To remove any other detergent, the mixture is dialyzed overnight a 4°C in 2L HEPES with Bio-Beads™ SM-2 Resin.

V-vesicle were made using the v-liposomes containing RB-dextran and mixing VAMP2 at a lipid-to-protein ratio of 200:1. The mixture is diluted 3-fold with 90 μM of HEPES to ensure the contents of the v-vesicle remains constant. V-vesicles were then dialyzed overnight similarly to the reconstituted t-lipids.

### Vesicle-to-Supported Bilayer Content-Release Fusion Assay

As described previously (Khounlo et al., 2021), a quartz slide along with a cover slip is hydroxylated by boiling in piranha solution composed of a 1:1 mixture of sulfuric acid and 30% hydrogen peroxide for 15 min. The cover slip and slide are rinsed in ddH2O 3 times to remove the piranha solution. To remove and residual acid, they are then sonicated for 30 min. After sonication, the slide and cover slip are rinsed once more with ddH2O and dried using nitrogen gas. The quartz slide and cover slip are assembled into microfluidic chambers using double sided Scotch tape to make each channel. The channels are then filled with t-lipids and incubated at 37°C for 2 h to ensure the t-lipids form a mobile and stable supported bilayer. The excess t-lipids in the channel are removed by flowing HEPES buffer into the channel.

Just as in our previous work (Khounlo et al., 2021), to image the slide, the slide is place onto the imaging stand of the microscope. Oil, with the same reflective index as our prism, is first added to the surface of our prism that will allow total internal reflection fluorescence microscopy (TIRFM). The prism is lowered onto the quartz slide. The incident angle of the exciting laser (532 nm) is adjusted to the proper imaging position. We then proceed with high resolution and real-time imaging. Our imaging area is 110 × 110 μm and we record at 20 ms time resolution. The viewing area is 512 × 512 pixels. The assay begins when the v-vesicles are flowed into the chambers at a speed of 50 μL/min and a total of 60 s of video is recorded. In assays preformed with cpx1 and the mutations of cpx1, the vesicles are incubated with the protein of choice for 10 min. The total sample entering the chambers has 250 nM if v-vesicles (total lipid concentration) encapsulating ∼90 μM of RB-Dextran. The 3-fold dilution causes the sample to contain approximately 3.75 nM of RB-Dextran in the total solution, which does not affect our measurements. The recorded events are then analyzed using a custom-build analysis software.

### Data Analysis

Our 60 s recordings were analyzed using an in-house MATLAB^®^ 2019 1) analysis software as previously described (Khounlo et al., 2021). Each video was analyzed frame by frame and each event’s fluorescence of RB-dextran was recorded by summing up the brightness of a 5 × 5 pixel area surrounding a central pixel at the center of the event. The event started with the immobilization of a vesicle. The data collected for large pore releases started as soon as 2D diffusion of the fluorophore was detected with the analyzer. The end of the event was after no more 2D diffusion was detected and the fluorescence returned to the baseline. This initial 2D diffusion resulted in a high spike in intensity and a slow decay back to the baseline over several seconds. Each event presented in this study was manually counted. Non-release events are defined as a vesicle that is immobilized on the supported bilayer for several seconds and results in an immediate increase in fluorescence and no decrease over that time period, after which, the vesicle suddenly disengages with the bilayer. These traces look like a sharp plateau and no slow decay back to baseline indicating that no pore was formed. We have also observed no substantial fluorescence decay due to photobleaching of our encapsulated dye at our time scales. These non-release events are too ambiguous to include in any data presented in this study. The large pore content release event traces were background-corrected by fitting the minimum baseline for all traces from a single recoding with a polynomial and then subtracting the polynomial from all traces.

## Data Availability

The raw data supporting the conclusion of this article will be made available by the author, without undue reservation.
